# Prognostic value of Multidetector Computed Tomography of cirrhosis

**DOI:** 10.1186/1471-2318-11-S1-A7

**Published:** 2011-08-24

**Authors:** A D’Andrea, A Reginelli, M Petrillo, F Iacobellis, S Cappabianca, R Grassi, L Brunese, A Rotondo

**Affiliations:** 1 Section of Radiology, Department Magrassi-Lanzara, Second University of Naples, Italy; 2 Department of Radiology, Health Science, University of Molise, Campobasso, Italy

## Background

Liver biopsy is an invasive test used to diagnose chronic liver disease and to assess the degree of hepatic inflammation and fibrosis. In recent years the accuracy of noninvasive tests has increased. The aim of this study was to evaluate whether the hepatic attenuation detected at triphasic MDCT was related to the degree of cirrhosis and allow the prognosis in human patients to be established. Afterwards we have also defined the parenchymal structural alterations and vascular changes detected by CT in an animal model of hepatic cirrhosis.

## Materials and methods

Multiphasic CT scans of 74 patients (24 healthy controls; 50 with liver cirrhosis, classified by Child-Pugh score) were retrospectively evaluated. Time-density curve of arterial, venous and late phases for each group of Child-Pugh score were calculated and compared between control and cirrhotic patients. In the experimental animal model with carbon tetrachloride induced cirrhosis, the CT findings and the histological features (optical and electron microscopy), were compared.

## Results

Compared to healthy controls, the attenuation in the arterial phase in the cirrhotic patients was increased, whereas the enhancement in the portal phase was decreased. The late phase showed a different hepatic outflow between the two groups, with high values in cirrhotic patients if compared with control group. A significant relationship between portal attenuation value and Child-Pugh score was found. Moreover, the enhancement percentage in the arterial phase was increased in the cirrhotic patient, balancing partly the modified hepatic inflow.

CT findings were confirmed in the animal models, in which an altered hepatic perfusion, especially in the late phase, was detected. The fluorescent medium contrast delayed washout was related to the parenchymal injury. It was demonstrated by optical microscopy that showed high grade fibrosis and change of normal lobular structure and by electron microscopy showing modified hepatic perfusion.

## Conclusions

The hepatic enhancement of portal and late phase detected at MDCT with multi-phasic scanning protocol is modified in cirrhosis. The MDCT could be a useful method of grading the disease.

The MDCT findings were related with hepatic cirrhosis grading, as observed in animal models. The decreased flow in portal phase and the delayed wash out depends on the modified parenchymal structure of cirrhosis.

